# Lattice Dynamics and Structural Phase Transitions in Eu_2_O_3_

**DOI:** 10.1021/acs.inorgchem.1c00708

**Published:** 2021-06-18

**Authors:** Jan Łażewski, Małgorzata Sternik, Paweł T. Jochym, Jochen Kalt, Svetoslav Stankov, Aleksandr I. Chumakov, Jorg Göttlicher, Rudolf Rüffer, Tilo Baumbach, Przemysław Piekarz

**Affiliations:** † Institute of Nuclear Physics, Polish Academy of Sciences, 31-342 Kraków, Poland; ‡ Laboratory for Applications of Synchrotron Radiation, Karlsruhe Institute of Technology, Karlsruhe 76131, Germany; § Institute for Photon Science and Synchrotron Radiation, Karlsruhe Institute of Technology, Eggenstein-Leopoldshafen 76344, Germany; ∥ ESRF-The European Synchrotron, Grenoble 38043, France

## Abstract

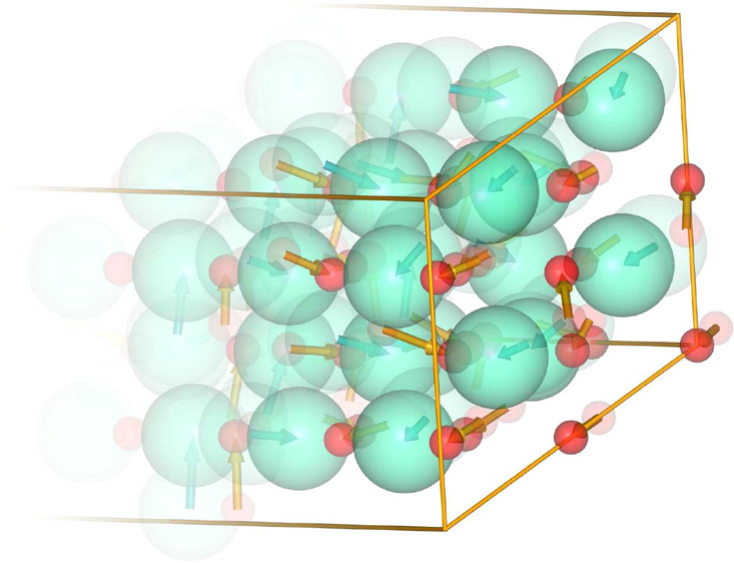

Using the density functional theory, we study the structural and lattice dynamical properties of europium sesquioxide (Eu_2_O_3_) in the cubic, trigonal, and monoclinic phases. The obtained lattice parameters and energies of the Raman modes show a good agreement with the available experimental data. The Eu-partial phonon density of states calculated for the cubic structure is compared with the nuclear inelastic scattering data obtained from a 20 nm thick Eu_2_O_3_ film deposited on a YSZ substrate. A small shift of the experimental spectrum to higher energies results from a compressive strain induced by the substrate. On the basis of lattice and phonon properties, we analyze the mechanisms of structural transitions between different phases of Eu_2_O_3_.

## Abstract 2

The phase transitions of rare-earth sesquioxide Eu_2_O_3_ are very complicated. Analyzing dynamical properties of this compound for experimentally observed C-, A-, and B-type phases and extracting key symmetry elements common for these phases, we describe transition mechanisms and their sequence. Our DFT first-principles predictions are supported by the nuclear inelastic scattering measurements.

## Introduction

Due to the high reactivity with oxygen, the most stable compounds of rare-earth (RE) elements are their oxides with a general formula R_2_O_3_, called sesquioxides, in which the RE ions (R) exist in the trivalent state.^1^ The unique physical and chemical properties of the RE sesquioxides such as the dense Kondo effect, heavy fermionic behavior,^2^ and high dielectric constants,^3^ just to mention a few, make them attractive from both scientific and technological perspectives. Europium oxide exists in three stoichiometric forms. At lower oxygen pressure, europium oxidizes first to the NaCl-type EuO and then to the spinel-type Eu_3_O_4_ before the stable sesquioxide is formed. The trivalent Eu^3+^ ions with the 4f^6^ electron configuration (^7^F_0_) have a total angular momentum *J* = 0 and *L* = *S* = 3.

At ambient conditions, Eu_2_O_3_ crystallizes in a cubic (C) structure and experiences structural transformations to monoclinic (B), trigonal (A), hexagonal (H), and cubic (X) phases with increasing temperature.^1,4^ Under pressure, the structural transition from the cubic C-type to the trigonal A-type phase, which starts at 5.0 GPa and finishes at about 13.1 GPa, is observed. This transition leads to a volume collapse of 9% at 8.6 GPa.^5^ The trigonal phase remains stable up to the highest experimentally feasible pressure. After release of the pressure, the trigonal phase transforms to the monoclinic phase.^5^ Also a direct phase transition from the C-type structure to the B-type monoclinic structure was observed at about 8.0 GPa, and the B-type structure was retained after the pressure was released, indicating that the monoclinic phase is metastable at room temperature.^6^ A pressure-induced phase transition from the monoclinic to trigonal crystal structure was observed at about 4.7 GPa.^7^ Finally, at ambient conditions, Eu_2_O_3_ may exist in a stable cubic form and in a metastable B-type structure similar to the Sm_2_O_3_ and Gd_2_O_3_ compounds. Both materials transform to the trigonal structure under a high pressure of about 5 GPa. The effect of pressure on R_2_O_3_ has been extensively studied by many research groups, and a review of the pressure-induced phase transitions of most sesquioxides is presented in ref (8).

The lattice dynamics of Eu_2_O_3_ was investigated by Raman scattering in the single crystal and powder samples at ambient conditions,^9,10^ and in nanocrystalline samples, both at ambient conditions and under hydrostatic pressure.^11^ As, in contrast to the bulk crystal, the Eu_2_O_3_ nanoparticles do not transform to the trigonal phase under high pressure, there are no Raman spectra measured for the A-type structure so far. When compared with other RE sesquioxides, the cubic phase of the europium compound systematically shows anomalously low Raman shifts (softening) for the middle-frequency oxygen vibrations.^10^ It was suggested that this anomaly results from the presence of oxygen vacancies (nonstoichiometry) in the crystal structure. To gain deeper insight, first-principles studies of the phonon properties of Eu_2_O_3_ are indispensable.

In the previous density functional theory (DFT) studies, the electronic properties of the cubic C-type and trigonal A-type phases were studied for different magnetic orders.^12^ The C → A structural transition was found near *p* = 5 GPa, which is in good agreement with the experimental data. A detailed investigation performed within the approach that combines the GW and local density approximation (LDA) + *U* methods found a strong effect of local Coulomb interaction *U* on the electronic bands.^13^ The stability of the cubic (C), trigonal (A), and monoclinic (B) phases of Eu_2_O_3_ was studied within the LDA + *U* method, and the sequence of the pressure-induced phase transitions C → A → B was predicted.^14^

In this paper, we study the structural and dynamical properties of the cubic (C), trigonal (A), and monoclinic (B) phases of Eu_2_O_3_ using the DFT approach. We examine the stability of these phases under pressure. The Eu-partial phonon density of states (DOS) obtained for the cubic structure is compared with nuclear inelastic scattering (NIS) measurements. Furthermore, we compare the calculated energies of Raman modes with the available experimental data for the cubic and monoclinic structures. Finally, we analyze the crystal structure changes and propose possible mechanisms of phase transitions between cubic, trigonal, and monoclinic structures.

## Calculation Method

The spin-polarized DFT calculations were performed using the projector augmented-wave potentials,^15,16^ the generalized gradient approximation in the Perdew–Burke–Ernzerhof (PBE) parametrization,^17^ and the following valence electron configurations 5s^2^5p^6^4f^7^6s^2^ and 2s^2^2p^4^ for Eu and O, respectively, as implemented in the VASP code.^18,19^ In this approach, all core electrons are treated fully relativistically. For valence electrons, we did not include spin–orbit coupling (computationally very demanding) since we checked that its influence on phonon dispersion relations of Eu_2_O_3_ is negligible; so they were treated scalar-relativistically. The strong local electron interactions were included within the DFT + *U* scheme,^20^ assuming the intraorbital Coulomb parameter *U* = 8.3 eV and the Hund’s exchange *J* = 0.77 eV on the 4f orbitals as in the earlier studies of EuO.^21,22^ This choice of parameters can be justified by the comparison with the experimental data. The electronic band gap of Eu_2_O_3_ obtained with a similar parameter *U* agrees very well with the measured value.^13^ The Hund’s exchange *J* weakly depends on the valency state; therefore, the selected value is appropriate also for Eu_2_O_3_. The energy cutoff for plane wave expansion was set to 520 eV.

The lattice parameters and atomic positions of the C-, B-, and A-type phases were optimized for supercells, which are 2 × 2 × 2, 1 × 3 × 1, and 3 × 3 × 2 multiplication of conventional cells of the cubic, monoclinic, and trigonal structures, respectively. All calculations were performed for *T* = 0 K and a ferromagnetic order of the magnetic moments of Eu atoms. A *k*-mesh of 4 × 4 × 4 points in the Monkhorst–Pack scheme^23^ was used for integration over the reciprocal space of cubic as well as trigonal structures and 2 × 2 × 2 points for the monoclinic phase. All structures were optimized with respect to the external pressure and atomic forces using the conjugate gradient technique with the energy convergence criteria set at 10^–7^ and 10^–5^ eV for electronic and ionic iterations, respectively. The maximum residual stresses were below 0.01 GPa for all simulated systems.

For the relaxed structures, the phonon dispersion relations as well as the total and element-projected phonon DOS were calculated using the direct method^24^ implemented in the PHONON software.^25^ In this approach, the Hellmann–Feynman forces generated on all atoms of the supercell by single-atom displacements from the equilibrium positions are used to determine the force constants and build the dynamical matrix. The phonon energies and polarization vectors were calculated by diagonalization of the dynamical matrix. To describe the longitudinal optic/transverse optic (LO/TO) splitting induced by macroscopic polarization, the static dielectric tensor and the Born effective charges were determined using density functional perturbation theory.^26^

## Theoretical Studies

### Crystallographic Structure

In the present study, we consider the cubic, trigonal, and monoclinic phases (traditionally marked with C, A, and B letters, respectively) of Eu_2_O_3_ observed experimentally.^4^ The cubic cI80 structure is the stable one for europium sesquioxide at ambient conditions. It is described by the *Ia*3̅ (206) space group. The rhombohedral primitive unit cell contains 8 Eu_2_O_3_ formula units (40 atoms). For the calculations, the cubic crystallographic cell with 80 atoms was used. The optimized lattice constant reads *a*_c_ = 10.959 Å and agrees well with the experimental value of 10.859 Å (after ref (12)).

The trigonal phase, described by the *P*3̅*m*1 (164) space group, has a primitive hexagonal unit cell containing one formula unit (five atoms). Our calculations were carried out in the 3 × 3 × 2 hexagonal supercell with 90 atoms. The relaxed lattice constants are *a*_h_ = 3.782 Å and *c*_h_ = 5.945 Å. The theoretical values obtained at pressure *p* = 6 GPa (3.749 and 5.788 Å), where the trigonal phase is stable, correspond well to the experimental values (at 5.72 GPa) of *a*_h_ = 3.719 Å and *c*_h_ = 5.770 Å.^27^ It is worth noting that the crystal density is significantly higher (about 10%) in the trigonal phase compared to the cubic structure, which fully agrees with the value of the volume collapse at high pressure reported in ref (5).

Finally, the monoclinic mS30 structure of the *C*2/*m* (12) space group and six formula units in the primitive unit cell was studied with the 1 × 3 × 1 supercell of 90 atoms. This phase is observed at high temperatures (above 1000 K) and can also be found as a metastable state at room temperature.^5^ By relaxation of the monoclinic structure, we found the lattice parameters *a*_m_ = 14.29 Å, *b*_m_ = 3.63 Å, and *c*_m_ = 8.89 Å and the monoclinic angle β = 100.14° similar to those obtained experimentally: *a*_m_ = 14.12 Å, *b*_m_ = 3.60 Å, *c*_m_ = 8.82 Å, and β = 100.02°.^9^ Comparing the crystal volumes optimized at ambient conditions, one should notice about 2% difference between monoclinic and trigonal structure volumes, which corresponds well to −1.6% volume collapse experimentally observed in the B → A phase transition^7^ and supports the first-order character with a displacive mechanism of this transition suggested by Atou et al. for the Sm_2_O_3_ compound.^28^

### Phonons

**Figure 1:**
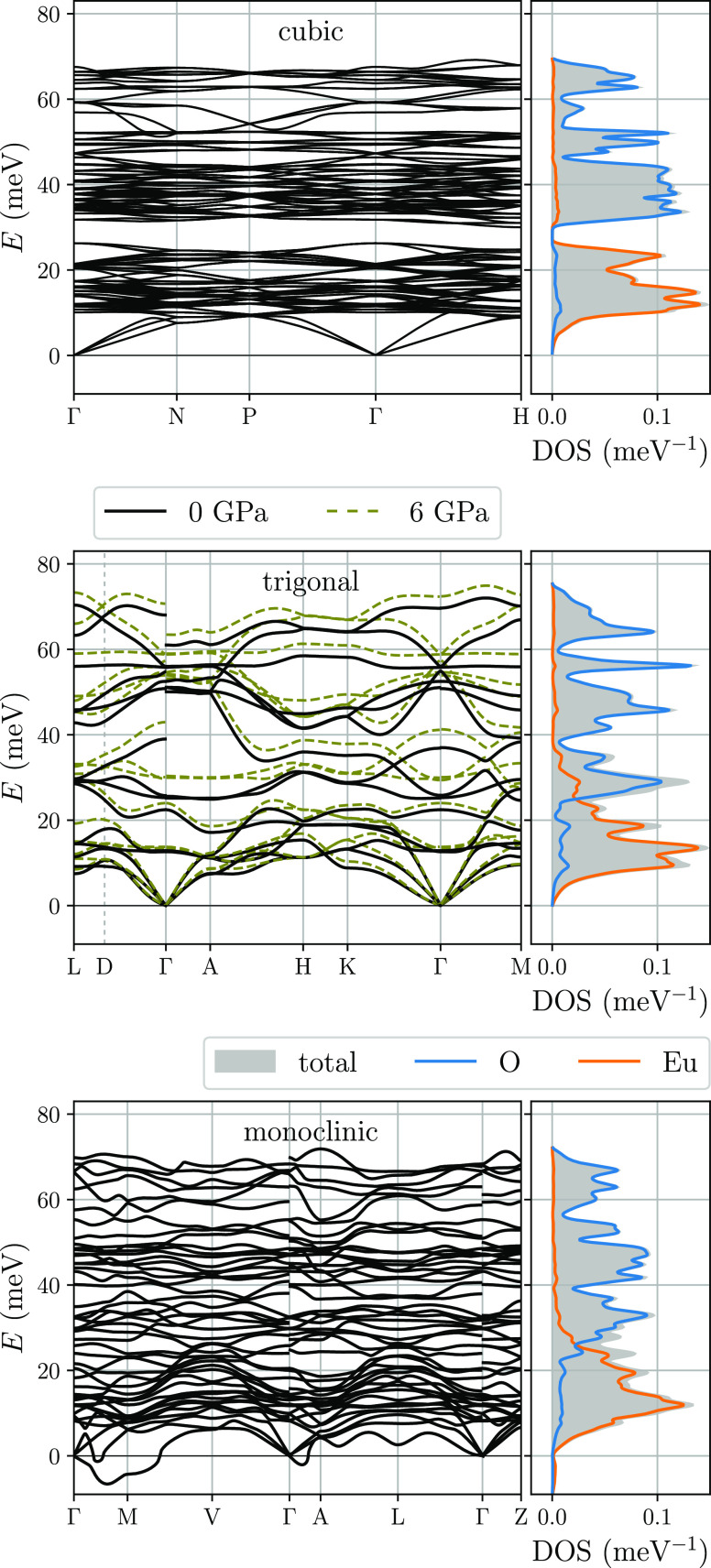
Phonon dispersion relations and phonon DOS in the cubic, trigonal, and monoclinic structures of Eu_2_O_3_.

For all optimized structures, the phonon dispersion relations and phonon DOS were calculated, and they are presented in Figure 1. According to the mass sequence, europium atoms occupy states of mostly lower energies, while phonons with oxygen contribution dominate at higher energies.

The cubic phase, which is stable at ambient pressure, has all phonon branches with real frequencies (Figure 1, left-top panel). The phonon dispersion relations are rather flat, which is an evidence of relatively weak interatomic interactions. The element-projected phonon DOS for this phase (color lines in right-top panel) are split and almost completely separated from each other with a narrow gap (∼5 meV) around 30 meV.

For the trigonal phase, which is not stable at ambient conditions, we present two different sets of phonon dispersions calculated at *p* = 0 and 6 GPa, which are depicted with solid and dashed lines, respectively, in the middle panel of Figure 1. The pressure dependence is clear as due to shortening of interatomic distances, bonds stiffen and phonon frequencies increase. Compared to the cubic phase, the number of phonon branches reduces because of the smaller number of degrees of freedom (one formula unit instead of eight in the cubic structure), and the dispersions of these branches are enhanced. Also, the LO/TO splitting, present at the Γ point, is more pronounced, and the highest energy accessible in the spectrum increases by a few millielectron volts in comparison to the cubic phase. These changes can be well understood by taking into account the significant increase of crystal density mentioned above (from 4.25 to 4.75 amu/Å^3^) in the same external conditions. The narrow energy gap in phonon DOS, observed for the cubic phase, closes and the partial DOS overlap slightly.

The phonon dispersion relations calculated for the monoclinic structure are presented in the lowest panel of Figure 1. In the primitive unit cell, there are 15 atoms (3 formula units) which leads to 45 nondegenerated phonon branches. Similar to the trigonal structure, the increase of the LO frequencies at the Γ point—connected with the macroscopic electric field generated by longitudinal displacements of atoms in different directions—causes discontinuities of the infrared-active modes. Along some directions of the reciprocal space, mainly around the *M* point, the lowest branch exhibits imaginary frequencies (plotted with negative values) reflecting the dynamical instability of this structure at low temperatures and ambient pressure. The calculations of phonon dispersion curves for the monoclinic structure at *p* = 10 GPa show similar soft-mode behavior, indicating that it cannot be stabilized only by pressure, and thermal effects play an important role in obtaining the stable or metastable B-type phase.

The Eu- and O-atom projected phonon DOS resemble that calculated for the trigonal structure. The vibrations of heavier atoms dominate in the low-frequency region up to 23 meV. In contrast, the vibrational energy of oxygen atoms can be divided into several distinct regions that occur at frequencies and intensities comparable to those in the trigonal phase. This is a result of the close structural relationship between the *C*2/*m* and *P*3̅*m*1 phases of Eu_2_O_3_.^9,29^ The B-type structure can be obtained by a slight lattice deformation of the A-type phase, leading to a splitting of 1*a* (*D*_3*d*_) and 2*d* (*C*_3*v*_) atomic positions into less symmetrical 2*b* (*C*_2*h*_) and 4*i* (*C*_*s*_) sites.^9^

### Zone-Center Phonon Modes

Group theory predicts 120 zone-center vibrational modes in the cubic C-type structure
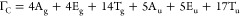
1where A_g_, E_g_, and T_g_ are Raman-active modes (R), T_u_ is infrared-active (I), and A_u_ and E_u_ are silent modes. T modes are also denoted as F modes. The E and T phonon modes are doubly and triply degenerate, respectively.

Regarding the monoclinic B-type structure, 45 zone-center vibrational modes are described by one-dimensional irreducible representations
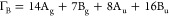
2The even (g) and odd (u) modes are Raman-active and infrared-active phonons, respectively.

Finally, the A-type structure has the following 15 zone-center vibrational modes
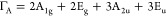
3where A_1g_ and E_g_ modes are Raman-active and the E_u_ and A_2u_ modes are infrared-active.

The zone-center mode frequencies (wave-numbers in cm^–1^) calculated using DFT, their irreducible representations (IR), and activities (R—Raman and I—infrared) are presented in Table 1. The calculated frequencies are compared with the available experimental data of the Raman modes obtained for the cubic and monoclinic structures.^9−11^

**Table 1:** Phonon Modes with Their Wavenumbers in cm^–1^, IR (Irreducible Representation), and Activities (R—Raman and I—Infrared) at the Γ Point in the Cubic (C), Monoclinic (B), and Trigonal (A) Phases of Eu_2_O_3_^a^

cubic					monoclinic					trigonal		
DFT	exp.^10^	exp.^11^	IR	act.	DFT	exp.^9^	exp.^11^	IR	act.	DFT	IR	act.
81.320			A_u_		66.016	73		B_g_	R	(p = 0 GPa)		
86.612			T_u_	I	66.995			A_u_	I	102.882	E_g_	R
92.634	94		T_g_	R	74.098			B_u_	I	181.178	A_1g_	R
93.709			E_u_		80.284	84		A_g_	R	204.863	E_u_	I
97.116	109		T_g_	R	94.785	98		B_g_	R	208.299	A_2u_	I
112.027			T_u_	I	96.565			A_u_	I	407.859	E_u_	I
115.535	119		A_g_	R	96.681			B_u_	I	423.705	A_1g_	R
121.585			T_u_	I	105.705	110		A_g_	R	442.687	A_2u_	I
128.096			T_u_	I	111.425	116		B_g_	R	450.793	E_g_	R
130.337	134		T_g_	R	116.110			A_g_	R			
139.500			T_u_	I	120.178			B_u_	I	(p = 6 GPa)		
140.698	145		E_g_	R	147.452	152		A_g_	R	110.069	E_g_	R
164.168			A_u_		160.756			B_u_	I	193.680	A_1g_	R
168.224			T_g_	R	165.173	176		A_g_	R	237.039	A_2u_	I
172.173			E_u_		193.372			B_u_	I	243.344	E_u_	I
172.643			T_u_	I	208.192	218		A_g_	R	432.852	E_u_	I
173.005	175		T_g_	R	219.847			A_u_	I	440.653	A_1g_	R
211.860			T_u_	I	227.579			B_u_	I	463.042	A_2u_	I
256.386			T_u_	I	239.846	246		A_g_	R	474.964	E_g_	R
274.184			A_u_		240.969	259		A_g_	R			
274.880			T_u_	I	249.971			B_u_	I			
277.061			T_g_	R	260.563			B_u_	I			
284.093			T_u_	I	261.324			A_u_	I			
285.673	289	266.4	T_g_	R	262.561	285		B_g_	R			
292.680	289	266.4	E_g_	R	323.499			A_u_	I			
304.218			T_g_	R	333.151			B_u_	I			
305.459			T_u_	I	348.358		354	A_g_	R			
313.276			A_g_	R	354.865	374		B_g_	R			
315.053			E_u_		363.248			B_u_	I			
315.672			T_u_	I	363.360			A_u_	I			
327.793			T_g_	R	378.339	394		B_g_	R			
330.202			E_g_	R	381.214	377		A_g_	R			
342.784			E_u_		389.317			A_u_	I			
348.414	339	336	T_g_	R	391.367	413		B_g_	R			
361.327			T_u_	I	410.883	424	409.7	A_g_	R			
373.941	385		A_g_	R	416.482			B_u_	I			
381.304			A_u_		446.008	465		A_g_	R			
381.566		380	T_g_	R	480.203			B_u_	I			
399.728			T_u_	I	509.404			B_u_	I			
407.908	425		T_g_	R	537.596	575		A_g_	R			
420.185			T_u_	I	538.824			B_u_	I			
458.867			A_u_		551.119	579		A_g_	R			
477.200		459	T_g_	R								
503.227			T_u_	I								
513.872			A_g_	R								
520.226			E_g_	R								
529.301			E_u_									
535.489	559		T_g_	R								

a For the trigonal structure, the values for *p* = 0 and 6 GPa are presented.

In the cubic structure, there are 22 Raman modes, but the number of peaks actually observed experimentally is much smaller because of the insufficient intensity and/or too small spectral resolution. For example, two Raman peaks with calculated frequencies of 285.7 and 292.7 cm^–1^ are reported as being of the *T*_*g*_ + *E*_*g*_ symmetry because these two peaks with different symmetry are measured at a coinciding frequency of about 289 cm^–1^ in the polycrystalline powder.^10^ In the nanocrystalline sample with the average particle size of 60–70 nm, the same peak is observed for a slightly lower frequency, 266.4 cm^–1^.^11^ In general, all measured values are adequately reproduced in our DFT calculations as shown in Table 1.

The comparative study of Raman spectra of R_2_O_3_ sesquioxides with the C-type crystal structure presented in ref (10) allowed to formulate some general relationships between Raman frequencies and the cubic lattice parameters. According to them, the frequencies of high-energy peaks of R_2_O_3_ decrease monotonically with the lattice constant. The only exception is Eu_2_O_3_, where the intermediate energy modes (from 280 to 400 cm^–1^) show an anomalous decrease of the frequency in comparison with the other R_2_O_3_ compounds. The authors formulated the hypothesis that this anomalous “softening” is related to the oxygen vacancies in Eu_2_O_3_. However, the measured frequencies agree well over the whole energy range with our values calculated for an ideal crystal. In view of the above, it is clear that the assumption of the oxygen deficiencies being a source of the anomalous softening is questionable.

Raman spectra measured for Eu_2_O_3_, Ga_2_O_3_, and Sm_2_O_3_ single crystals with B-type monoclinic structure do not exhibit any anomalous behaviour.^9^ All frequencies of Raman-active modes are in good agreement with the calculated data.

To the best of our knowledge, there is no Raman spectroscopy data for the trigonal structure of Eu_2_O_3_, but the Raman modes measured for other A-type sesquioxides are arranged just as in our calculations: two bending vibrations of low-frequency between 100 and 200 cm^–1^ and two stretching vibrations occurring at a higher frequency region between 400 and 450 cm^–1^.^9,30,31^ Under pressure, the Raman modes of the trigonal structure shift to higher frequencies. In Table 1, the frequencies of the zone-center modes calculated for the trigonal structure at *p* = 0 and 6 GPa are presented.

## Experimental Results

In order to verify the *ab initio* calculations performed for the cubic phase, the theoretical results were compared with the Eu-partial phonon DOS of a polycrystalline Eu_2_O_3_ film obtained from NIS. Exposed to air, metallic Eu rapidly oxidizes forming a mixture of cubic and monoclinic phases of Eu_2_O_3_ with a high concentration of oxygen vacancies. Moreover, the hygroscopic nature of Eu_2_O_3_ favors the formation of Eu hydrates and hydroxides, which further contaminate the sesquioxide. Using a commercially available Eu_2_O_3_ powder, the Eu-partial phonon DOS was determined by NIS on the Mössbauer-active isotope ^151^Eu of europium.^32,33^ Sufficient details, however, of the sample characterization were either not presented or confirmed the problems described above. Therefore, to investigate the pure cubic sesquioxide phase, a 20 nm thick Eu_2_O_3_ film was deposited on a YSZ(001) substrate in the ultrahigh vacuum system^34^ located at the Nuclear Resonance Beamline ID18^35^ of the ESRF-The European Synchrotron in Grenoble, France. Prior to Eu deposition, the substrate was annealed at 925 K for 60 min at a pressure below 3.0 × 10^–9^ mbar. A metallic Eu foil enriched to 97% in the Mössbauer-active isotope ^151^Eu, supplied by the Oak Ridge National Laboratory (USA), was sublimated from an effusion cell with a molybdenum crucible for producing a steady flux of Eu atoms at the rate of 6.0 Å/min. During deposition of europium, the substrate was kept at 823 K, and high-purity (99.9995%) molecular oxygen was supplied into the growth chamber at a pressure of 1.0 × 10^–6^ mbar, precisely controlled via a leak valve. To ensure complete oxidation of the metallic Eu, the film was annealed for 60 min under the deposition conditions. In order to protect the Eu_2_O_3_ film from further oxidation, it was covered at room temperature by an 8.0 nm thick Nb layer.

**Figure 2:**
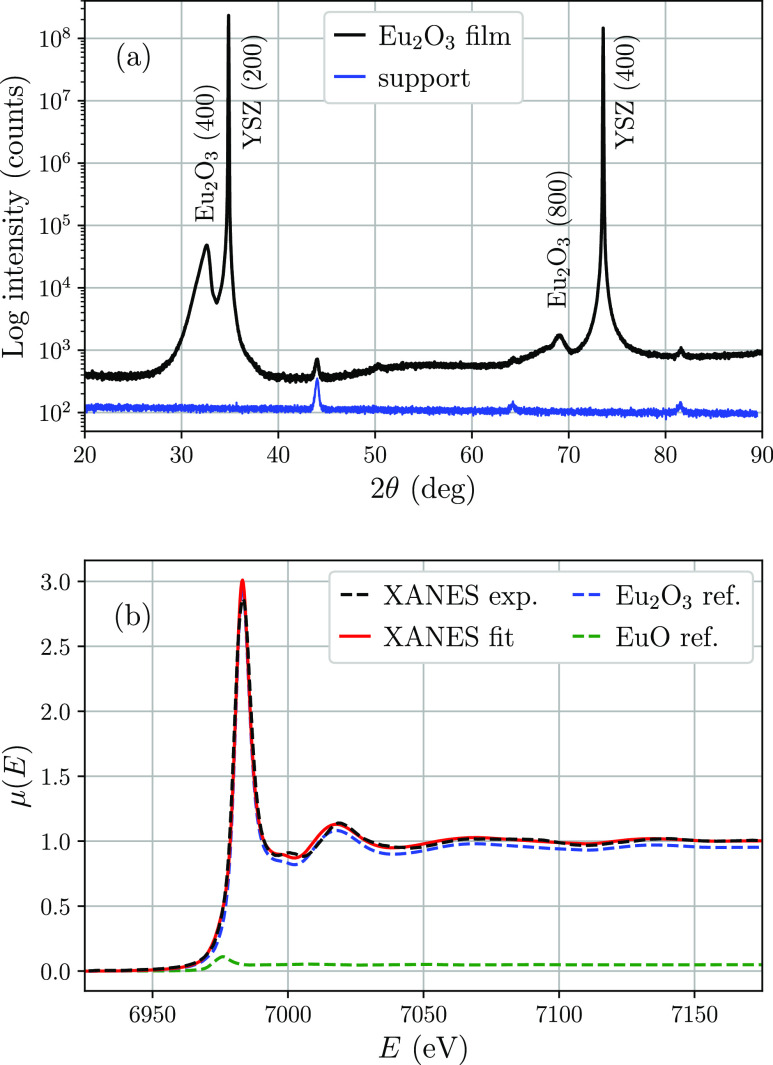
(a) XRD scan on the investigated sample and the supporting plate along with the identified peaks. (b) XANES data and simulated curve assuming the model described in the text. Reference data are given in their relative weights, i.e., 5% and 95% for EuO and Eu_2_O_3_, respectively.

The sample was characterized by X-ray diffraction (XRD, Cu K_α_ line using a Rigaku SmartLab instrument) and X-ray absorption spectroscopy on the Eu L_3_ absorption edge of Eu (6977 eV) performed at the SUL-X beamline of the Synchrotron Radiation Source at Karlsruhe Institute of Technology in Germany. The ATHENA and ARTEMIS program packages, from the IFFEFIT software,^36^ were used for data reduction and modeling. The XRD scan shown in Figure 2a confirms the formation of the cubic Eu_2_O_3_ phase with a lattice constant *a* = 10.80 Å, which is ca. 0.5% smaller than the bulk value *a* = 10.86 Å. The scan on an empty support plate (without a sample) revealed the origin of the additional peaks present in the XRD data. The oxidation state of Eu was determined by comparison of the experimental X-ray absorption near edge structure (XANES) data on the film with a reference Eu_2_O_3_ powder sample and EuO films^21,22^ measured in the same experiment. The obtained XANES data are plotted in Figure 2b and show a distinct difference between the position of the absorption L_3_ edge of Eu in EuO where the Eu atoms, similar to the metallic Eu, exhibit an oxidation state of Eu^2+^ and in Eu_2_O_3_ where the Eu atoms are in the Eu^3+^ state. By using the least-squares method, the XANES data of the film were fitted by a linear combination of the two reference samples with the ratio between them being a fit parameter. The result is plotted in Figure 2b by a solid/red line and indicates the presence of 5.0% EuO in the investigated film. However, the contribution of monoxide is hardly visible as a small shoulder of the strong sesquioxide absorption peak. Most likely, this phase is formed at the film/substrate interface, where the control of the oxygen concentration is challenging.

**Figure 3:**
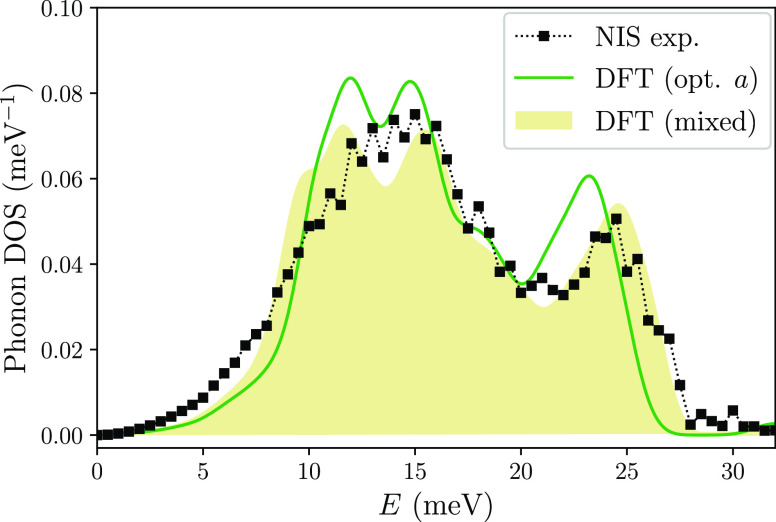
Eu-partial phonon DOS of the cubic phase of Eu_2_O_3_. The experimental data are compared with DFT results calculated for the optimized unit cell (solid line) and mixed (95% C-phase Eu_2_O_3_ + 5% EuO) spectrum obtained with the fixed experimental lattice constant (filled shape).

The ^151^Eu-partial phonon DOS was obtained^37^ from the energy dependence of the probability for nuclear inelastic absorption^38,39^ of X-rays with energy 21.5414 keV with an energy resolution of 1.1 meV (full width at half maximum)^40^ measured at room temperature at the Nuclear Resonance Beamline ID18. The film was illuminated at a grazing angle of about 0.15° by a focused X-ray beam with dimensions, vertical × horizontal ≈ 10 μm × 100 μm. Figure 3 compares the experimentally obtained DOS with the theoretical results. Compared with the DOS obtained for the optimized lattice constant (solid/green line in Figure 3), the experimental spectrum is slightly shifted to higher energies due to the compressive strain induced by the substrate. In order to verify this assumption, we calculated the DOS for the cubic structure with the lattice parameter estimated by the XRD measurement, relaxing only atomic positions and combining the calculated Eu-partial DOS of Eu_2_O_3_ with EuO. The relative weight of both contributions is fixed to the results obtained by the XANES study, i.e., 95% Eu_2_O_3_ and 5% EuO. To account for the broadening of the phonon spectrum features, which originate from the finite energy resolution and phonon scattering at crystal imperfections, the *ab initio*-calculated DOS was additionally convoluted with a Gaussian profile of fwhm = 6 meV. The resulting spectrum shows a very good agreement of the peak positions and the cutoff energy in relation to the experimental data. The higher number of phonon states below 10 meV in the experimentally obtained DOS can be attributed to interface-specific vibrational modes often present in thin films,^22,41^ which are not considered in the *ab initio* calculations performed for a bulk crystal.

## Structural Phase Transitions

The total energies obtained after optimization at *p* = 0 read −47.2563, −47.0005, and −47.0456 eV per formula unit for cubic, trigonal, and monoclinic phases, respectively, which confirm that the most stable phase is the cubic one. The quite small difference between the energies of trigonal and monoclinic phases can be assigned to the displacive phase transition between them. It agrees with the previous theoretical and experimental values of energy differences between these two phases.^14^

The pressure-induced phase transition between the cubic and trigonal phases of Eu_2_O_3_ is of the first order.^5^ Indeed, our calculations for pressure *p* = 6 GPa show that the respective enthalpies are equal to −44.249 (cubic) and −44.282 eV (trigonal) per formula unit. The previous *ab initio* study also supports this result showing the crossing of enthalpies around 5 GPa.^12^

In the first-order phase transition, the crystal structure changes discontinuously and the atomic displacement pattern can be very complicated. In the case of Eu_2_O_3_, where *Z* changes by a factor of 8 and a significant (≈10%) collapse of the volume is observed, it is quite hard to establish a correspondence between the cubic and trigonal structures since the majority of 40 atoms in the primitive *Ia*3̅ unit cell are significantly displaced. To describe this rearrangement in a systematic way, we have split the problem into three stages: relative orientation of cell vectors, deformation of the cubic supercell, and finally, tuning of atomic positions. We started from two general observations: (i) in the trigonal structure, the threefold axis along the main diagonal of the cubic structure should be preserved and (ii) the number of atoms in the system should not change. These assumptions limit possible transformations to the rotation aligning the threefold axis in both structures, scaling along this axis while keeping the atomic density constant, and rigid translations. The values of the parameters of these transformations (angles, scaling factors) could be determined by minimizing the sum of squares of distances between corresponding atoms in both structures. In general, this function has a very complicated shape and multiple local minima; thus, it is difficult to minimize it by classical gradient-type methods. Therefore, we have used a multistage procedure employing the first step of the genetic algorithm searching the whole parameter space for possible valleys, the basin-hoping algorithm to select the deepest one, and simulated annealing to find the best local minimum followed by standard least-squares minimization of the distance function. All above steps were monitored and visually inspected with custom-built visualization program using ASE and NGLview python libraries^42,43^ and JupyterLab environment.^44^

**Figure 4:**
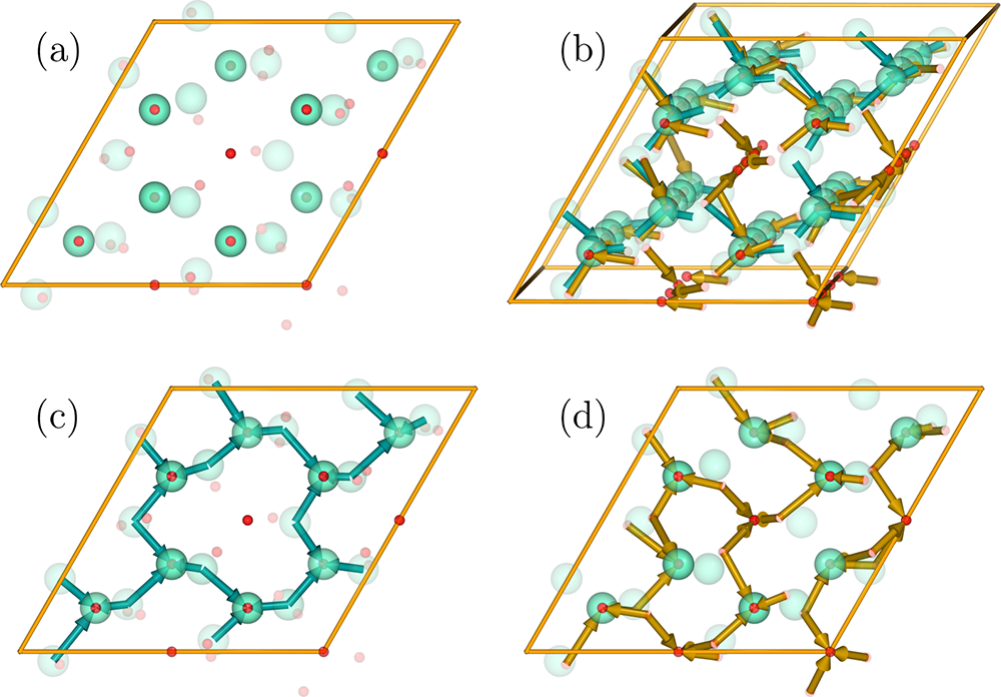
2 × 2 × 4 supercell of the trigonal structure of Eu_2_O_3_ (solid spheres) superimposed on the positions of corresponding atoms in the cubic structure (translucent spheres): (a) positions of atoms; (b) rearrangement vectors marked with arrows; (c,d) displacements of Eu (in green) and O (in red) atoms separately. The [111] direction of the cubic structure is aligned along the *c* vector of the trigonal structure. Live 3D version of this figure is available in the Supporting Information as Figure S1.

The final relationship between the structures is depicted in Figure 4 in several views along the *c*-axis of the trigonal structure, showing atoms of the cubic structure as translucent spheres and atoms in the positions from the trigonal structure as more solid spheres. The relationship between structures is illustrated by arrows connecting the corresponding atoms. Subfigure (a) shows just the atoms, panel (b) shows, slightly tilted for better clarity, all relationships between atoms, and panels (c) and (d) show the relationships between Eu and O atoms separately. The structural relationships depicted in Figure 4 are rather difficult to comprehend on the flat static image. Therefore, we have prepared a live 3D version of this figure, accessible in the Supporting Information as Figure S1, which can be interactively rotated, zoomed, and so forth with any modern web browser.

Generally, atomic displacements in the C → A phase transition are substantial. The mean square displacement (MSD) of Eu and O atoms, ignoring the contribution of volume collapse and overall cell deformation, reads 1.386 and 1.803 Å^2^, respectively. Each four Eu layers (perpendicular to the threefold axis) with very pronounced atom movement are separated alternately by the other four Eu layers with smaller shifts. Looking along the threefold axis, a careful observer can see in the trigonal structure the removal of Eu atoms (occupying in cubic phase 8*b* Wyckoff’s positions) from the centers of Eu hexagons in favor of O atoms and creation of oxygen chains instead of Eu–O–Eu–O zig-zags characteristic for the cubic structure. In the C phase, all Eu–O distances are the same; however, after the phase transition (in the A phase), the oxygen atoms can be divided into two groups: (i) O1, located in 2*d* (*C*_3*v*_) Wyckoff’s positions, closer to europium atoms and (ii) O2, occupying 1*a* (*D*_3*d*_) sites, positioned between Eu–O1 dimers. The effect is not very strong; however, a difference in distances of O2 and O1 to the nearest Eu atom exceeds 10%. During the phase transition, Eu–O1 dimers rotate, ordering themselves along the hexagonal axis into Eu–O–Eu–O chains, while “lonely” oxygen atoms, O2, overcome the barrier between two neighboring Eu atoms and locate themselves in the oxygen chains in the center of the Eu hexagons.

**Figure 5:**
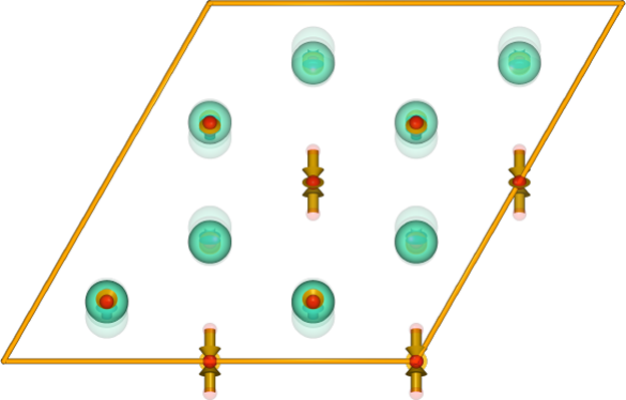
Relationship between the trigonal and monoclinic structures of Eu_2_O_3_. The main displacements are limited to changing positions of oxygen atoms (in red) on the axis of hexagonal europium (in green) rings. Live 3D version of this figure is accessible in the Supporting Information as Figure S2.

In the A → B phase transition, the situation is completely different. The MSD of heavy Eu atoms drops down more than 1 order of magnitude (0.068 Å^2^) in comparison to the C → A transition. Also, the MSD of oxygen atoms is significantly smaller (0.281 Å^2^). Inner Eu–O1 distances shorten slightly due to O1 movement along the Eu–Eu line parallel to the hexagonal axis. In turn, O2 atoms move perpendicularly—out of straight oxygen chains (Figure 5).

**Figure 6:**
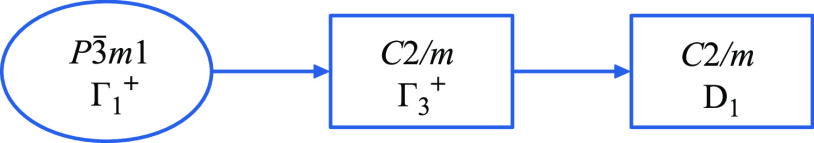
Diagram of irreducible representations and order parameters for the trigonal-to-monoclinic phase transition derived with *Get_irreps* package from the Bilbao Crystallographic Server.^45−47^

Having both trigonal and monoclinic structures fitted well with each other, we were able to define a transformation matrix TM
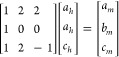
4and a relative shift of origin [1/2, 0, 0]. We obtained Wyckoff’s positions splitting
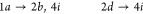
5confirmed by the group-theoretical predictions.^48^ Finally, using Bilbao Crystallographic Server package *Get_irreps*,^45−47^ we drew a diagram (Figure 6) of physically irreducible representations and order parameters for the A → B phase transition with transformation matrix TM. The symmetry relation between these phases reveals a serial cascade of continuous changes of the crystal structure, which are coupled with the shear deformation causing a displacive phase transition. However, the transition has a first-order character with a finite change in volume. The mentioned shear deformation originates from the rise of the monoclinic angle and can be linked with slight softening of acoustic phonon branches around the Γ point visible in Figure 1. We found that this elastic instability is strongly coupled with the lowest optical mode at the Γ point. Therefore, it can be expected that by lowering the external pressure, one can obtain from the trigonal structure a metastable monoclinic phase through the displacive first-order phase transition rather than the cubic structure through the order–disorder transition, which usually has a significantly higher energy barrier. The situation changes at high temperatures, where thermal fluctuations start to play an important role.

## Conclusions

We have performed a theoretical and experimental study of the structure and lattice dynamics of Eu_2_O_3_ sesquioxide. Using the first-principles DFT approach, we calculated the structural parameters and phonon spectra in the cubic, trigonal, and monoclinic structures. The close structural relationship between the monoclinic and trigonal structures is reflected in the calculated partial Eu and O phonon DOS, which are very similar for both phases. In the cubic phase, the partial DOS of europium and oxygen atoms are separated by a narrow gap, which distinguishes the lattice dynamics of this phase from the others.

A good agreement between the calculated and measured frequencies of the Raman modes was found for the C- and B-type structures. The Raman frequencies of the A-type structure, which were unknown so far, are also presented.

The calculated phonon DOS for the cubic phase was verified experimentally. The ^151^Eu-partial DOS was measured at room temperature for a 20 nm thick Eu_2_O_3_ film using NIS. The formation of cubic Eu_2_O_3_ was confirmed by XRD, while X-ray absorption spectroscopy unveiled the presence of ca. 5% EuO. The experimental phonon DOS showed a good agreement with the DOS calculated for a C-type structure assuming 0.5% lattice compression, most likely induced by the substrate.

We also analyzed the phase transitions observed in Eu_2_O_3_: C → A and A → B. We developed a numerical procedure for searching the atomic rearrangement during the phase transition. Analyzing structural changes, in particular, atom rearrangement, in the cubic-to-trigonal (C → A) phase transition, we discovered creation of monoatomic oxygen chains along the threefold axis. Substituting O2 atoms of the trigonal structure with other elements of the periodic table, one can design R_2_O_3_-like functional material with pressure/temperature-controlled optical or transport (conductivity) properties.

## Supporting Information Available

Live 3D figure presenting the relationship between cubic and trigonal structures (HTML)
Live 3D figure presenting the relationship between trigonal and monoclinic structures (HTML)

## Supplementary Material

ic1c00708_si_001.htm

ic1c00708_si_003.htm
