# Supplementary material for: Lattice Dynamics and Structural Phase Transitions in Eu2O3
Source: Inorg Chem. 2021 Jun 18;60(13):9571–9. doi: 10.1021/acs.inorgchem.1c00708 (PMC8277167; doi:10.1021/acs.inorgchem.1c00708)
Supplement: Supplementary file 2 — ic1c00708_si_003.htm [file ic1c00708_si_003.htm]

Eu2O3 Hexagonal vs. Monoclinic Structure


Lattice dynamics and structural phase
transitions in Eu2O3  
Supporting Information

Jan Łażewski,† Małgorzata Sternik,† Paweł T. Jochym,† Jochen Kalt,‡,¶
Svetoslav Stankov,‡,¶ Aleksandr I. Chumakov,§ Jorg Göttlicher,¶ Rudolf Rüffer,§
Tilo Baumbach,‡,¶ and Przemysław Piekarz†

† *Institute of Nuclear Physics, Polish Academy of Sciences, Kraków, Poland*  
‡ *Laboratory for Applications of Synchrotron Radiation,
Karlsruhe Institute of Technology, Karlsruhe, Germany*  
¶ *Institute for Photon Science and Synchrotron Radiation,
Karlsruhe Institute of Technology, Eggenstein-Leopoldshafen, Germany*  
§ *ESRF-The European Synchrotron, Grenoble, France*

  
  

**Figure S2:** The relationship between monoclinic (transparent atoms) and hexagonal (solid atoms)
unit cells in Eu2O3.
The arrows indicate atomic displacements between structures.

The figure can be rotated by mouse dragging and panned by dragging with
ctrl key pressed. The center of rotation can be changed by clicking on the atom.
Zooming is possible by shift-dragging or scrolling. Resizing the view is done
by dragging triangular handle in bottom right corner, and full-screen mode is
accessed by clicking the "two-arrows" icon in the top right corner.
